# Application of Sorbent-Based Extraction Techniques in Food Analysis

**DOI:** 10.3390/molecules28247985

**Published:** 2023-12-07

**Authors:** Natalia Drabińska, Monika A. Marcinkowska, Martyna N. Wieczorek, Henryk H. Jeleń

**Affiliations:** Faculty of Food Science and Nutrition, Poznań University of Life Sciences, Wojska Polskiego 31, 60-624 Poznan, Poland; natalia.drabinska@up.poznan.pl (N.D.); monika.marcinkowska@up.poznan.pl (M.A.M.); martyna.wieczorek@up.poznan.pl (M.N.W.)

**Keywords:** food analysis, pesticide residues, food contaminants, volatile compounds, flavor, food matrix

## Abstract

This review presents an outline of the application of the most popular sorbent-based methods in food analysis. Solid-phase extraction (SPE) is discussed based on the analyses of lipids, mycotoxins, pesticide residues, processing contaminants and flavor compounds, whereas solid-phase microextraction (SPME) is discussed having volatile and flavor compounds but also processing contaminants in mind. Apart from these two most popular methods, other techniques, such as stir bar sorptive extraction (SBSE), molecularly imprinted polymers (MIPs), high-capacity sorbent extraction (HCSE), and needle-trap devices (NTD), are outlined. Additionally, novel forms of sorbent-based extraction methods such as thin-film solid-phase microextraction (TF-SPME) are presented. The utility and challenges related to these techniques are discussed in this review. Finally, the directions and need for future studies are addressed.

## 1. Introduction

In recent years, substantial developments in analytical chemistry, particularly in separation sciences and sample preparation techniques for food analysis, have been observed. Food is one of the most challenging matrices due to its complexity, non-homogenous state and high contribution of polar compounds [1,2]. The complexity of food analysis is a result of matrix parameters and the nature of analyzed compounds. This makes the sample preparation process very challenging and highly matrix dependent [3,4]. Compared to air or water, which are relatively simple matrices, food can vary in terms of basic constituents and compounds that may potentially interfere with analytes. Therefore within various foods, we can find those which consist of only one main type of macroconstituent (i.e., edible plant oils) or those with all the main groups of macroconstituents—proteins, carbohydrates and fats—in a single product and their mutual proportions can vary to a great extent. Moreover, basic matrix constituents may play an integral part in food processing (i.e., coffee and other thermally processed foods where the Maillard reaction plays a crucial role in product development). Importantly, many of the compounds of interest can be “hidden” in a matrix, occurring in both free and bound form; therefore, very often, an additional sample preparation step involving hydrolysis is required [5]. Another challenge in food analysis is very often an ultra-low concentration of compounds, which requires additional preconcentration steps and highly sensitive analytical approaches.

The sample for analysis should be representative and consistent, and a problem with some food matrices is their lack of homogeneity. Therefore, chopping or blending is often used as the first step in sample preparation. It has to be kept in mind that in the homogenization process, enzymatic reactions can be induced, which may influence both the matrix and the profile of compounds analyzed. Some foods are in the form of emulsions (i.e., milk, butter, and mayonnaise), which will influence the behavior of analytes in such a matrix (i.e., their partition coefficients, which would influence headspace analysis). The turbidity of some products (i.e., fruit juices) should also be taken into consideration. The physical properties of the matrix (mainly its viscosity) will have special importance in the analysis of aroma compounds and their release from the matrix. When hydrocolloids such as gelatine, starch or pectin are compared in terms of the release of flavor compounds, the partition coefficients may vary between them, as a result of both the entrapment of flavor molecules and interaction with gel components [6].

Sample preparation steps in food analysis should provide appropriate clean-up and elimination of interferences, and achieve sufficient preconcentration for reliable quantitation of compounds of interest. With the developments in selective detection methods (i.e., high-resolution mass spectrometry, HRMS, tandem mass spectrometry, MS/MS), or enhanced selectivity in separation (i.e., comprehensive two-dimensional gas chromatography, GC × GC and two-dimensional liquid chromatography, LC × LC), the influence of the matrix can be minimized, but cannot be neglected. The number and diversity of compounds in food make the process of their extraction more complex. Various extraction techniques, such as liquid–liquid extraction (LLE) and, more recently, deep eutectic solvents, were proposed to extract different analytes in food matrices, and they were well characterized in other excellent reviews [7,8]. Many compounds with similar physicochemical properties coexist in one product, thus all of them will be extracted similarly. Therefore, the extraction method has to be selective to certain compounds. Sorbent-based extraction (SBE) has developed in recent decades as an alternative to conventional extraction techniques, which use many more solvents and do not meet the Green Analytical Chemistry (GAC) requirements [9]. The development of new techniques of SBE and new sorbent enabled cheaper, more cost-effective, selective as well and more environmentally friendly sample preparation methods. Taking into account the rapid development of the SBE techniques, this review aims to characterize and summarize the SBE methods used in food analysis, underlying their benefits and limitations, new devices and solutions as well as pointing out the need for future studies. To date, there are many reviews available focusing on only one type of extraction technique, like solid-phase microextraction (SPME) [10,11]. In the present work, we wanted to present a plethora of techniques which are based on the sorption phenomenon.

## 2. The Introduction to SBE Methods

Among all SBE methods, solid-phase extraction (SPE) is the most often used technique in the food chemistry field, due to both its long history as well as the broad fields of application resulting from the selectivity of sorbents used in SPE. The ability of solid surfaces to bind organic compounds has been known for years and the first experiments were performed with activated carbon, which was used among other applications in the closed-loop stripping analysis with all its benefits and drawbacks (i.e., irreversible adsorption, varying recoveries, etc.). The developments in SPE in the 1960s and 1970s and the use of Amberlite XAD resins, Tenax, and finally bonded silica cemented the popularity of SPE [12,13]. In particular, modification of silica with alkyl or aryl groups (C2, C8, C18, diol, cyanopropyl, phenyl) resulted in the success of this technique in food analysis. In 2002, a new chapter in SPE applications opened, especially in pesticide residues and veterinary drugs analysis, when Anastassiades et al. [14] developed an effective method of the extraction of especially polar and basic compounds. This method is named QuEChERS (for Quick, Easy, Cheap, Effective, Rugged and Safe) and it is dispersive SPE (d-SPE), revolutionized sample preparation in pesticide residue analysis. The success of QuEChERS in terms of popularity is reflected in many published reports and also in a database for recoveries for over 650 pesticides and metabolites (www.eurl-pesticides-datapool.eu (accessed on 10 November 2022)). The purpose of dispersive SPE clean-up is the removal of organic acids, sugars, lipids, and pigments, mainly carotenoids and chlorophylls. The most often analyzed samples using QuEChERS for which AOAC methods are developed are fruits and vegetables, even those with fats, waxes, and pigments [15].

The second popular SBE method is solid-phase microextraction (SPME), which was developed by Pawliszyn in 1990 [16]. The idea behind integrating the sorption of analytes into fused silica fiber coating and subsequent desorption into the injection port of a gas chromatograph (GC) in syringe-like fiber/holder simplified sampling and analysis of volatile organic compounds (VOCs). Due to the complexity of food matrices, SPME is usually performed from the headspace (HS-SPME). This approach enables the analysis of compounds in solid matrices without solvents and in a shorter time. However, recently, some attention was focused on the direct extraction from liquid food samples since it was proven that it is a more effective way to extract polar compounds [17]. Additionally, the next direction of SPME, still not sufficiently explored for food analysis, but used for biological fluids is the combination of SPME with LC–MS [18]. Another new era is using SPME with an ambient MS, without chromatographic separation [19]. This area of SPME application is still under investigation; nevertheless, results achieved so far indicate that it might be a technique of choice in the future. Several methods using needle-based extraction were developed afterwards, among them solid-phase dynamic extraction (SPDE), where sorbent is on the inner wall of a needle mounted in a gas-tight syringe, needle-trap devices (NTD) or in-needle capillary adsorption trap (INCAT) [20].

Extraction techniques based on similar principles followed SPME development, such as stir bar sorptive extraction (SBSE) or high-capacity sorbent extraction (HCSE), which also found many applications in food analysis. Molecularly imprinted polymers (MIPs) are used frequently in the sample preparation of food. Very high selectivity is possible by tailoring MIPs for specific compounds, enabling highly sensitive and selective analyses of veterinary drugs and other contaminants in food [21]. Novel techniques, such as microextraction methods based on the use of magnetic nanoparticles as supports, gradually find applications in food science, especially in the area of contaminant analysis. The applications include but are not limited to the analysis of melamine, polycyclic aromatic hydrocarbons (PAHs), and phthalates in milk, tea and edible oils as well as herbicides, pesticides, synthetic dyes in tea, vegetables, wines and chili powder. Moreover, this approach was used for aflatoxins and ochratoxin A analysis in milk, cereals, rice and corn [22]. As the majority of these studies were carried out within the last 10 years, there are perspectives on the increasing role of magnetic nanoparticles in sample preparation for food analysis.

Considering the frequency of use, various foods and matrices tested and the broadest range of analyzed compounds, two SBE techniques prevail in food analysis—SPE and SPME; therefore, the next two subchapters will be devoted to applications of these techniques.

## 3. SPE in Food Analysis—Main Fields of Applications

### 3.1. Lipid Analysis

Lipids form the fundamental constituents of food that perform necessary structural, functional and biological properties. Lipids play an important role in cells of the biological systems but also have considerable influence on eligible food quality factors, e.g., aroma, color, structure, texture, and taste sensation. Commonly, consumer acceptance of food is strictly related to the quality of its lipids [23].

**Figure 1:**
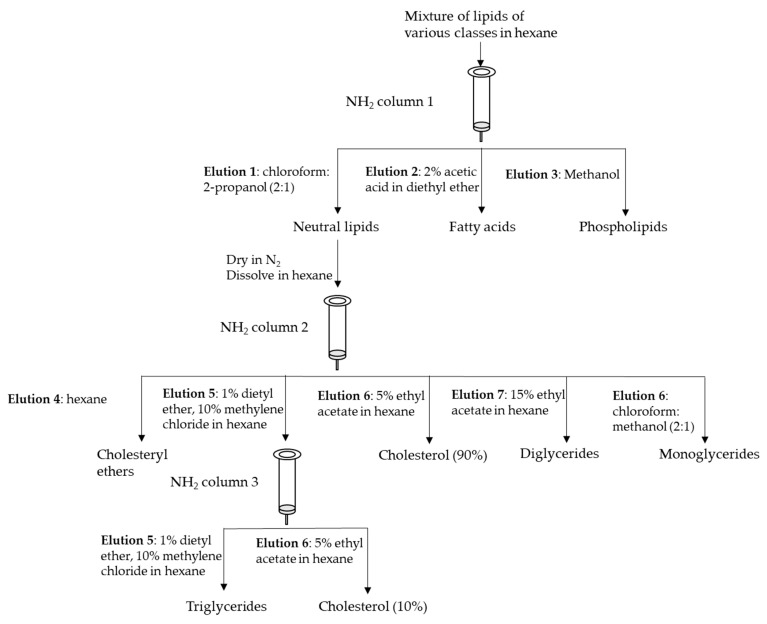
Fractionation of lipid classes using NH_2_ SPE columns. Based on [24].

In general, single or dual organic solvent extraction, liquid–liquid extraction (LLE) and SPE are mainly used to extract, and then fractionate lipids. The main advantage of the utilization of SPE with a properly selected sorbent enables isolating particular lipid classes. A basic scheme for the fractionation of lipid classes proposed by Kaluzny et al. [24], which utilizes an NH_2_ column for the fractionation of a lipid mixture into fractions of different polarity, can be seen in Figure 1. SPE is commonly used for analytes enrichment, especially when they are present in a sample at a low concentration, such as eicosanoids, steroid hormones and fatty acid esters of hydroxy fatty acids. Accurate and proper fractionation of lipid classes in muscle foods analysis is required to achieve an authentic fatty acid profile appropriate for a given fraction. A traditional approach for muscle lipid separation utilized the SPE method; however, the main problem was with the neutral lipid fraction, which contains a certain amount of phospholipids. The further development of that method, changing the type, proportion, and volume of used solvents as well as the aminopropyl minicolumn size, contributed to reducing phospholipids in the first fraction to almost undetectable.

The SPE method is also supportive in the determination of free fatty acids (FFAs) in beer in a reliable and simple way. The presence of FFAs in beer considerably affects consumer acceptance of beer quality, in particular the foam stability and flavor [25]. Analysis of FFAs by chromatographic methods (GC, HPLC) requires a sample pre-treatment step. SPE with aminopropyl cartridges, in comparison to the previously used method (steam distillation and LLE) is faster, more reproducible, less complicated and demands a minor volume of organic solvents. Moreover, the emulsion creation issue resulting in analyte loss is eliminated. Additionally, the SPE technique is appropriate to determine a wide variety of fatty acids (differing with the number of carbons in the chain) in contrast to other extraction methods like SPME or SBSE [25].

Lipid autoxidation is a chain reaction which consists of three stages: initiation, propagation, and termination. The compounds obtained are chemically active and participate in further free radical processes [26]. SPE is commonly used to control the content of potentially harmful compounds in the oxidation process. Regardless of the analytical technique used (GC–MS, HPLC–MS, HPLC–MS/MS) for monitoring the deterioration of lipids, the sample preparation step is required and relies on a double SPE process which applies C18 and silica cartridges. Determination of 3-monochloropropane-1,2-diol and its esters, not only in edible oils but also in fats and food with high content of fats, is performed using SPE [27]. The new approach assumes the application of d-SPE with Si-SAX-PSA and Z-sep+-PSA sorbents as a sample pre-treatment step. d-SPE has many advantages compared to solvent-based methods—for instance, shorter clean-up times, lower costs, lower amounts of solvents required, and less waste. Further, utilizing optimized d-SPE enhances precision and recovery and reduces matrix effects [27].

Phytosterols are found in cells of plant material and play a crucial structural function in maintaining the stability of biological membranes. Plant sterols exist as free or bound (esters, glycosides) species and they have proven health-beneficial properties, decreasing low-density lipoprotein (LDL) cholesterol [28]. SPE is suitable to separate covalently bound plant sterols from lipid extracts (prior and posterior hydrolysis process) in purification procedures or to enrich the phytosterols fraction from other unsaponifiable lipids. SPE can be utilized to obtain polar and non-polar fractions, which should be processed and analyzed one by one [29].

Vitamins A, D, E, and K are gathered into clusters of fat-soluble vitamins. They represent essential micronutrients with a wide spectrum of important biological influences on human health [30,31,32,33]. As a result of their specific chemical structure, they are highly prone to several factors such as light sensitivity, oxygen access, pH value conditions, and temperature. The stability of vitamins makes an extraction process a critical step in further analysis. SPE with a silica column is commonly used for the analysis of fat-soluble vitamins in fruits, vegetables, meat, cereals, herbs, spices, seeds, seasoning blends, and baby food matrices [34].

### 3.2. Mycotoxins

Recently, the presence of mycotoxin contamination in food, considering both adverse effects on human health and economic losses resulting from spoiled products, has received attention worldwide [35]. Mycotoxins occur as native contamination in food matrices, and they can be synthesized by numerous fungal species before crop harvest, or during the storage process in poorly preserved food. The consumption of plant- or animal-originated products which contain mycotoxin contaminations can lead to serious diseases (headaches, abdominal ache, diarrhea, vomiting) or even increase the risk of cancer development due to the carcinogenic, mutagenic and teratogenic properties of mycotoxins [36]. Therefore, reliable mycotoxin analysis methods are necessary. The determination of mycotoxins in food usually relies on three main detection methods—HPLC with fluorescence detection, diode array detection (DAD) or tandem MS. Capillary electrophoresis, colorimetric detection, enzyme-linked immunosorbent assay (ELISA), GC, and thin-layer chromatography form a group of analytical approaches that are less frequently used. In any case, the key stage in the analysis of mycotoxins is the sample preparation process. Various techniques have been utilized so far like LLE, immune-ultrafiltration, pressurized liquid extraction, dispersive liquid–liquid microextraction (DLLME), and QuEChERS. SPE, applied individually or in combination with previously mentioned procedures, is commonly used as a regular method of mycotoxin extraction [37]. Microwave-assisted extraction utilizes microwave energy to increase the efficiency of rapid heating of organic solvents which interact with the sample in the base of the partition coefficient of the desired analyte. Although this method has many advantages (fast, more environmentally friendly due to cut-off organic solvents, higher extraction yield), SPE must be applied as an enrichment stage [38].

Ochratoxins belong to the class of toxins that are synthesized by heterotrophic fungi belonging to the genera *Aspergillus* and *Penicillium* (*Penicillium verrucosum*, *Aspergillus ochraceus* and *A. niger*, *A. carbonarius.*) [39]. These carcinogenic, immunotoxic, nephrotoxic and teratogenic compounds have been detected in food of animal origin, especially in pork and poultry, and are able to accumulate in the kidneys, liver, pancreas, and blood. At lower concentrations, mycotoxins have been observed in muscles, adipose tissue, and eggs, which makes the clean-up procedure considering the complexity of these tissues/fluids very complex. SPE with C18 columns method was used as a purification step followed by LC–MS/MS, enabling rapid determination of ochratoxins with high selectivity and specificity in animal tissues and other matrices [40].

The *Aspergillus* genus is an example of a fungal species which produces toxic secondary metabolites called aflatoxins. Aflatoxin B1 is considered the most important mycotoxin among that class due to high toxicity [41]. Aflatoxin M1 is a principal oxidation product of B1, which is resistant to heat treatment. Aflatoxins are predominantly present in cereals, nuts, and their products [41]. Inferior environmental conditions and development of the country may contribute to increasing the concentration of M1 aflatoxin in milk and dairy products. To analyze this type of mycotoxins in milk and dairy food matrix (goat milk, powder milk, whole, skimmed cow milk, yoghurt), online SPE with HPLC coupled with a fluorescence detector was applied [42]. Commonly, online SPE uses an ultra-HPLC (UHPLC) system with an injection loop and a ten-port switching valve (load or inject set) consisting of a chromatographic column and SPE with a C18 sorbent cartridge. There are many advantages of online SPE compared to traditional (offline) SPE methods, with high throughput being the main benefit [42].

Recently, an increase in the content of mycotoxins in vegetables and fruits was observed, in particular in dried figs, dried vine fruit, dried apricots, plums, and dates. Pressurized liquid extraction is a convenient method for coupling with an online SPE and detection system. Online automated SPE with a C18 cartridge in addition to offline traditional SPE ensures improved precision and sensitivity of determination of mycotoxins in trace amounts in the abovementioned matrices [43]. For the analysis of mycotoxins in food (corn flour, beer, milk powder, peach seed), a new approach based on the use of dispersive micro-SPE (d-µ-SPE) with zirconia nanoparticles as a dispersant has recently been used [38]. d-µ-SPE enables carrying out a single separation with an enrichment stage for the extraction and determination of aflatoxin B1, fumonisin B1, ochratoxin A, ochratoxin B, T-2 toxin, and zearalenone with crucial advantages such as reduced time, lower amounts of organic solvent, and less sorbent wear when compared to traditional SPE. Moreover, the desirable extraction yield causes high selectivity, acceptable reproducibility and, as a consequence, low limits of detection (LOD) [38].

Cereal grains can be readily contaminated by fungi responsible for toxins synthesis. The most dangerous mycotoxins for human health present in infected corn or wheat are fusarium toxins, aflatoxins, and ochratoxins, which are formed under proper conditions (moisture, temperature) [35]. The majority of the commercially available SPE cartridges are not adequate for the clean-up of various types of mycotoxins and often special columns are designed for specific sets of toxins. An SPE stationary phase (multi-walled carbon nanotubes) has been used for the extraction of fungal toxins. The main advantage of a newly applied SPE absorbent is its suitability for fast one-step purification for a wide range of mycotoxins. In addition, pre-treatment of the sample by multi-walled carbon nanotubes SPE cartridges has satisfactory recovery and, together with the use of UHPLC–MS analysis, forms a rapid, reliable and sensitive approach for the determination of a broad spectrum of mycotoxins [44].

### 3.3. Pesticide Residues

The development of agriculture in recent years has led to the use of pesticides that are sprayed on crops mainly for economic purposes. It is estimated that the use of these chemicals contributes to the protection of approximately 30% of crops by deterring pests and insects [45]. The use of pesticides not only increases crop yields but also involves risks to human health due to the extensive use of these resources without legal restrictions. Residues of pesticides can be detected in soil for several decades and effectively reduce its quality. Furthermore, many pesticides show bioaccumulation and biomagnification in concentration, which is harmful to human health [46]. Pesticide residue may be found in a wide range of food products (e.g., fatty animal-origin foods like eggs, fish, meat, milk, and poultry [47,48], and plant origin-food such as citrus essential oil, fruits and vegetables, and wines) which is an analytical challenge considering the pesticide contaminants in the complex matrix are present usually in low concentrations [49]. Focusing on the analysis of multi-residue pesticides in plant samples, it can be noticed that they have a wide range of matrix properties that cannot be omitted by choosing the optimal method for sample preparation (acidity, basicity, neutral, polar, non-polar). There are many techniques for extracting pesticides—liquid–liquid microextraction, LLE, liquid-phase microextraction, SPE, SBSE, supercritical fluid extraction, and QuEChERS—with d-SPE as the most often used one method [50]. The conventional method used to detect and quantify the number of pesticides in samples is based on chromatographic methods (GC or HPLC) with various detectors [51], but the sample preparation process is usually matrix specific.

The cinnamon used in medicine and as a spice is grown in a hot and humid climate, which makes it particularly prone to bacteria, fungi, and pests. Application of herbicides may cause not only high productivity but can also generate residues in cinnamon bark. Spices such as black pepper, paprika, and turmeric were reported as matrices that are difficult to analyze and induced a suppression effect as a result of complex chemical composition (essential oils, fatty acids, pigments, sugars) and excess water [52]. To eliminate co-extracted compounds, d-SPE with diverse types of sorbents was proposed. Primary–secondary amine (PSA) sorbents can be successfully used to reach substantial retention of fatty acids, organic acids, pigments and sugars instead of C18, which is applied to maintain the hydrophobic nature of the sample. Graphitized carbon black sorbent is effective against colored compounds like chlorophyll but it keeps other targeted composites. Satisfactory results for the analysis of spices are obtained by the combination of d-SPE with repetitive d-SPE with HPLC–MS/MS [52].

Apple, broccoli, shallot, and tea are examples of polyphenol-rich food products which may contain pesticide residues. Polyphenol compounds cause analytical problems as a result of interferences at the ionization step. The necessary step for determining pesticides in food with a high content of polyphenols is, therefore, clean-up. The sample pre-treatment stage included the precipitation of polyphenols by d-SPE with polyvinylpolypyrrolidone (PVPP) before dilution of the sample to eliminate the influence of the matrix. This made it possible to analyze 8 neonicotinoid insecticides and the further optimization and use of UPLC–MS/MS led to the determination of multiple classes (e.g., neonicotinoid, an organophosphate) of 20 different pesticides with a wide range of polarity. A high yield of the sample purification and reduced matrix effects were obtained without significant dilution of the cleaned-up fractions. This technique in combination with ESI-HPLC–MS/MS provides sufficient recoveries and satisfactory limits of quantification (LOQ) values to a wide range of pesticide residue [53].

To increase the production efficiency of olive oil and other vegetable oils, pesticides are commonly used that eliminate or reduce adverse factors for crops. There is much research on the detection and determination of pesticides in complex matrices like vegetable oils. The analysis of vegetable oils for pesticides is difficult due to a large number of co-eluting compounds. SPE with a alumina column or a C18 sorbent is commonly used for that purpose. Lately, this technique has been subjected to some modifications with the utilization of magnetic nanoparticles and MIPs as a new type of sorbents. As a result of the accessibility of novel sorbents, d-SPE is the most popular method so far, used to purify vegetable oil samples before GC or LC analysis [54].

Pesticides used for the protection of wines can be also found in the final product. The range and type of pesticides licensed for use in wine making and their maximum residue levels are determined by law regulations and differ between countries. Optimal conditions for the SPE application for red and white wine samples include the use of hydrophilic–lipophilic balance (HLB) sorbents and a mixture of acetonitrile and methanol as solvents. Extracts were injected into UPLC–MS/MS, which enabled the determination of 50 pesticide residues with a LOQ below 1 ng/mL for 48 of them [55]. Grapevines are prone to a wide range of pests (fruit worm, grapevine moth, vine mealybug) and fungal infections (black mold, grey rot, downy mildew, powdery mildew), which is why they have to be protected by utilizing regulated pesticide formulations. The clean-up stage was performed using d-SPE with PSA sorbent to remove the effects of the matrix. However, if a tandem MS is available, a sample purification step is less crucial [56].

It is not only foods of plant origin that are exposed to the presence of pesticide residues, since pesticides were also found in fish such as catfish, rainbow trout and shellfish like shrimps. Analysis of pesticides in aquatic species meat requires a clean-up step which is based on a widely applied technique, QuEChERS—d-SPE with primary–secondary amine (elimination of interferences such as fatty acids, organic acids, pigments) and C18 sorbents (cleansing from non-polar-constituents like lipids and waxes). Currently, new methods of purification of the sample are studied based on a modification of the sorbent bed, e.g., using multi-walled carbon nanotubes, zirconium-based sorbents or anhydrous salts such as MgSO_4_. The purified samples were analyzed by LC–MS/MS [57].

Honey provides a lot of desirable medical properties and it is an analytical challenge due to the complex matrix composed of numerous different types of compounds (amino acids, enzymes, essential oils, flavonoids, organic acids, phospholipids, sugars, sterols, and vitamins) [58]. Pesticides which can transfer into nectar or those that accumulate in the environment are the main pollution of honey. It is possible to determine pesticide residue in a honey sample by utilizing the GC or LC technique; however, in the sample pre-treatment step among which d-SPE with polymerically bonded, ethylenediamine-N-propyl (PSA) sorbent and acetate buffer dominate. It was observed that the purification process with PSA sorbent does not enable sufficient purification efficiency for the honey extract. To remove interferences and maintain the analyte concentration of the honey extracts, the d-SPE method is used with a complex sorbent composed of MgSO_4_, florisil, C18, PSA, Z-Sep+ sorbent, and citrate or acetate buffer. The optimal method of purifying a sample of honey turned out to be the use of mixed sorbents varying in clearance mechanism PSA (pH ionic exchanges) with Z-Sep+ (hydrophobic, Lewis acid interactions), MgSO_4_, and acetate buffer. The QuEChERS sample preparation technique with modifications enables the analysis of over 200 pesticides in honey [59].

### 3.4. Environmental Contaminants in Food

In addition to the mycotoxins and pesticide residues described above, food is exposed to more adverse factors such as environmental contaminants. This type of pollution could arise naturally (weathering of the earth’s crust, combustion) or it can be introduced artificially by humans (industrial uses or as undesired side products of industrial processes, combustion). Climate change contributes to the increase in the occurrence of phenomena such as cyclones, droughts, floods, forest fires, landslips, and storms and consequently to the increase in environmental contaminants. Animals are able to accumulate environmental impurities, incorporating them into the food chain and ultimately also into food consumed by humans. Metals, especially heavy metals (cadmium, lead, mercury), arsenic, brominated flame retardants, dioxins, furans, polychlorinated biphenyls, and PAHs, create a group of environmental contaminants. These compounds have an undesirable effect on human health and they are capable of causing chronic diseases or damaging internal organs [60].

Cereal and cereal derivative products are an important element in the human diet; however, cereal-based foods are also carriers of arsenic, especially if it comes from a location where the natural concentration of this element is high or it is contaminated. Maize, rice and wheat crops are therefore exposed to arsenic; moreover, plants do not use this element and are capable of bioaccumulation, which can harm the potential consumers. The most toxic varieties of arsenic are arsenite (As(III)) and arsenate (As(V)); therefore, the qualitative and quantitative determinations are focused on them. Speciation analysis of this element is complicated as a result of its relatively low ionization ability, monoisotopic character, and polyatomic interferences. The sample preparation step before analysis using the SPE is extremely important because it greatly improves the analysis results of cereal-based products such as tortillas by reducing interferences from the matrix [61].

Antimony, chromium, cobalt, copper, nickel, selenium, silver and thallium are slightly less harmful to human health compared to arsenic. SPE is utilized to obtain satisfactory resolution and to concentrate analyte level [62]. To determine the content of metals in food samples (e.g., seafood, water, and vegetables), different varieties of SPE are applied (offline, online, d-SPE); however, nowadays, magnetic SPE is considered the most convenient to use. Currently, sorbents based on magnetic nanoparticles attract attention due to numerous advantages (omission of the centrifuging/filtering step, high resistance against harsh factors, e.g., pH, temperature, substantial versatility in shape, size and sorption properties). The use of magnetic nanoparticles results in a greater efficiency in enriching the sample in the analyte, and consequently lower LOD and satisfactory reproducibility [62].

Polybrominated diphenyl ethers and polychlorinated biphenyls can be transferred to food from the environment. These organic compounds have a high persistence and bioaccumulation ability, thus enriching the food chain with an increasing amount of undesirable impurities. Intake of edible fishery and aquaculture products is associated with an increased risk of taking polybrominated diphenyl ethers and polychlorinated biphenyls [57]. These pollutants cause many negative effects on human health, thus their control is required, especially for seafood. Analysis of animal tissues is a challenge due to trace amounts of the analyzed compounds, and the need to eliminate interferences caused mainly by lipids, which leads to a significant reduction in the sample volume. The efficient process of purifying samples of biological tissue origin after accelerated solvent extraction uses sequential SPE (d-SPE as well as tandem SPE). For this purpose, various sorbents, e.g., silica gel, alumina, and Florisil, with modification were applied [63].

Flame retardants are a class of compounds containing emerging halogenated flame retardants, organophosphate flame retardants, and polybrominated diphenyl ethers. These compounds were added to many industrial products (building materials, electronics, foams, furniture, textiles) to prevent the fire from spreading. In the last decade, it has been discovered that despite their good operation during a fire, they are also extremely toxic and persistent in an environment where they accumulate and, therefore, their use has been banned. Flame retardants have been detected in air, soil, water and living organisms, and consequently also in food [64]. In the determination of these compounds, pigments, and lipids most often interfere, which can be eliminated during the sample cleaning step. The most commonly used technique for this purpose is the d-SPE with C18, Z-SEP or PSA sorbent. This technique has been successfully applied to bacon, chicken eggs, chicken thigh, lean beef, lean pork, mussels, prawn, salmon, smoked salmon, and tuna [64].

Vegetables are highly vulnerable to the absorption of organic impurities that can accumulate via the root system, especially when they are irrigated with recovered wastewater or sewage sludge from wastewater. Bioaccumulation depends on the nature of the contamination and the part of the plant, e.g., in the root (carrot, potatoes, turnip), the compounds with the anionic, neutral and cationic structures are most often accumulated while in the leaves (chard, lettuce, spinach) this order is reversed. Environmental contaminant accumulation influences crops and has potential adverse health effects (cancer and metabolic disorders, reproductive damage). Plant absorption is evaluated with particular emphasis on the analysis of antioxidants, disinfection by-products, flame retardant compounds, perfluoroalkyl compounds, personal care products, pharmaceuticals, and plasticizers [65]. As a result of these compounds that are present in trace amounts, an appropriate extraction method is required (focused ultrasound solid–liquid extraction, matrix solid-phase dispersion, pressurized solvent extraction ultrasound-assisted extraction), after which the concentration of the target analyte is necessary. The most commonly used technique for clean-up and enrichment samples is SPE or d-SPE with a polymeric sorbent applying an ion-pair reactant [65].

Chloramphenicol is a popular antibiotic, which was isolated in 1947 from *Streptomyces venezuelae*. A wide spectrum of activity against pathogens (Gram-positive bacteria, Gram-negative bacteria, and some of *Chlamydia* and *Rickettsia* species) and high efficiency lead to use against bacterial diseases in both animals and humans [66]. Unfortunately, chloramphenicol residue in edible animals enters the food chain. Routine determination of this compound is carried out by ELISA and chromatographic methods. Before determination by utilizing chromatography techniques, it is necessary to pre-clean the sample with SPE with magnetic molecularly imprinted polymers as a sorbent [66].

### 3.5. Processing Contaminants

As mentioned earlier, food can be contaminated by various paths and from different sources. Impurities arise during processes and thermal reactions as well as cleaning, disinfecting, sterilization and other preservation techniques. The analysis of processing contaminants is demanding because they most often occur in trace amounts in complex matrices, hence a sample pre-treatment stage is required to concentrate the analyte and eliminate interference from the matrix [67].

PAHs are widely known as carcinogenic compounds that are primarily produced during the thermal treatment of food (combustion, roasting, and smoking of organic matter). The most known compound in this class is benzo[a]pyrene, which is classified in category 1 according to the International Agency for Research on Cancer. Any smoked or thermally processed food may contain PAHs even in roasting raw materials like coffee beverages or dark beer. To clean the samples of coffee and dark beer, silica-gel cartridges were used; however, recovery was low in contrast to alumina-N SPE cartridges, which turned out to be suitable for this matrix. For the determination of PAHs, GC–MS, LC with a variety of detection methods (UV, FLD, MS) and, currently gaining recognition, supercritical fluid chromatography with carbon dioxide, which is suitable for low-polarity analytes and provides a shorter analysis time, were applied [68].

Edible oils are consumed in large quantities as part of our diet. Through the lipophilic nature of edible fats and their origin, they are highly exposed to toxic PAH pollutants. The main problem in the analysis is a matrix that consists of high-molecular-weight compounds (triglycerides); therefore, it is necessary to separate the analyte from the matrix and adequately clean the sample. To achieve a satisfactory sample, which can be analyzed by GC–MS/MS, the d-SPE was used. A modern multi-walled carbon nanotube sorbent was applied for selective extraction, which replaced the habitually used time-consuming techniques [69].

Meat, particularly smoked, is subjected to strict controls, using the SPE method. A recent study indicated a deficiency in the optimal extraction methods for different bovine tissues because of the unsatisfactory sensibility of the methods. Sufficient recovery values, LOD and LOQ were obtained by utilizing matrix solid-phase dispersion extraction before sample pre-treatment by SPE with a nucleoside silica C18 sorbent. The results obtained enable the monitoring of food samples because the method provides sufficient sensitivity below the permitted levels [70].

Derivatives of furan (2-methyl furan, 2-furaldehyde, 5-methyl-2-furaldehyde) form a group of organic heterocyclic compounds called furans. Nevertheless, in the literature, the term “furans” also refers to polychlorinated dibenzofurans, which commonly appear with dioxins as environment impurities. Typically, furan derivatives are not found naturally in food and are formed during fermentation, pathogen disinfection or preservation (pasteurization, thermal processing or UV light treatment). The presence of furans was found in a wide diversity of foods such as apple cider, bakery products, brandies, bread, coffee, fruit juices, honey, tequilas, vinegar, and wines. For the determination of furan derivatives, the SPE technique with polymer-based LiChrolut EN cartridges was used to purify the sample, as well as to isolate and concentrate the VOCs from liquid samples. The high diversity of sorbents creates many possibilities for extraction from lipophilic and hydrophobic matrices, which enables obtaining a satisfactory LOD [71].

Acrylamide is formed as a result of thermal processes (baking, grilling, frying) in foods with a high carbohydrate content based on the Maillard reaction mechanism [72]. As reported by the International Agency for Research on Cancer, acrylamide has several potential adverse health properties (carcinogenic, genotoxic, mutagenic); therefore, it is subject to specific controls and law regulations. This organic compound is present in various food matrices such as potatoes and coffee but a close relationship between its quantity and processing is observed. Acrylamide has many properties (polar molecule, hydrophilic properties, low molecular weight, low volatility) that make its analysis difficult by using regular analytical techniques. A particularly problematic case is the extraction of acrylamide with water because, at the same time, interferents are also extracted. To eliminate interferences, SPE is used as a regular sample clean-up step in the determination of acrylamide in coffee, French fries, grilled asparagus and potato chips. The majority of the sample preparation stage requires the use of two or more classic SPE. For potato samples of a strongly hydrophilic polymer, unique HLB cartridges were used; and for grilled asparagus samples, d-SPE with alumina Al_2_O_3_ sorbent was suitable [73].

### 3.6. Flavor Analysis

Flavor is an extremely important sensory factor that largely influences the choice and acceptance of food by consumers [74]. The great challenge in this field of analytical chemistry is the fact that there are enormously large differences in concentrations (high to traces) of compounds responsible for the taste or aroma. The crucial step for qualitative and quantitative determinations of flavor compounds is sample extraction and preparation. Traditionally the sample preparation for the analysis of flavor compounds, depending on the nature of the product and odorants, involves distillation methods, simultaneous distillation/extraction, and vacuum distillation to avoid the formation of artefacts, but also sorbent-based methods. The SPE technique fulfils many requirements because the ambient temperature of extraction prevents artefact formation, is robust and is not time-consuming. SPE eliminates the creation of emulsions and reduces the consumption of organic solvents. Another advantage is the possibility of using a wide range of sorbents with different properties. SPE has been effectively applied in the separation of flavor compounds from different food matrices [75].

Flavor compounds of tea arise from primary precursors amino acids/carbohydrates, carotenoids, glycosides, isoprenoids, lipids, and polyphenols during enzymatic oxidation and hydrolysis, non-enzymatic degradation and oxidation, and the Maillard reaction [76,77]. The aroma of the tea is caused by the quality of the plant, the climate in which the tea is grown (location, growing conditions) and the production process. To analyze over 160 VOCs in tea infusions, it was necessary to use comprehensive sample preparation methods: SPE, solvent-assisted flavor evaporation (SAFE) and SPME. SPE a silica cartridge provided aroma enrichment and a clean-up step mainly for compounds of lower volatility (benzyl alcohol, hexanoic acid, phenylacetaldehyde) in contrast to SPME, which is more sensitive with highly volatile compounds (acetaldehyde, dimethyl sulfide) [76].

The flavor of the wine is influenced by many components ranging from raw material, selected yeast, fermentation process and storage and ageing of the wine [78,79]. The key stage in the analysis of wine is extraction, aimed to remove impurities originating from the matrix, the isolation of specific VOCs or the concentration of analytes present in trace amounts. SPE with C18 and XAD-2 resin sorbents has been commonly used for the extraction of the glycosidically bound parent compound in grapes and wines. Excluding limitations of using the SPE method to isolate VOCs, SPE was found to be an appropriate technique to determine 12 higher alcohols, esters, and acids in wines, which are VOCs produced by yeast during fermentation [78]. During the analysis of wine samples, particular emphasis should be placed on the fractionation of extracts by the use of silica sorbents. Selective elution of VOCs by solvents differs in polarity, making it possible to analyze β-damascenone, esters, and sulfur compounds [78]. Determination of VOCs at trace levels (ng/L) in complex matrices such as red wine with satisfactory precision still provides a challenge for analytical chemistry. Currently, the optimal method described for the extraction of aroma compounds in wine samples is SPE with a C18 cartridge, which is considered a well-established sample pre-treatment technique. Moreover, the SPE method enables preconcentration and isolating the target analyte in free or bounded form and it is effective against interferences [80].

In a variety of honey samples to date, over 600 different VOCs have been determined, which illustrates how complex and difficult the matrix is. The efficiency of the entire analytical process depends on the selection of the extraction method, which is extremely important because the sample obtained has to be representative in comparison to the starting product. As an appropriate method for the extraction of VOCs from honey, SPE with a divinyl benzene-N-vinyl pirrolydone cartridge can be successfully used [81].

Beer is another example of a complex mixture of over 100 organic chemical compounds that belong to different classes but the most important from the sensory point of view are aldehydes, esters, higher alcohols, diacetyl and thiols. Apart from a few exceptions, aldehydes have pleasant aromatic green leaf, fruity and grassy notes. Thiols, on the other hand, are introduced together with the raw material and can be transformed at the stage of fermentation. Additionally, they act as antioxidants and retain the aroma of the beer. Therefore, beer can be considered a demanding matrix for analysis due to its complexity and also because of the reactivity of these organic compounds. SPE with C8-H_2_PO_3_ (thiols) and silica-COOH (aldehydes) is useful to enrich the sample and obtain more signals of target compounds [82].

Phenolic compounds are commonly found in foods of plant origin (e.g., beer, cereals, cider, cocoa, fruits, legumes, nuts, tea, vegetables, and wine) [83]. Phenols have a great influence on nutritional values and, above all, on the sensory quality of food. The phenolic content in food products is associated with astringency and bitter taste. Determination of phenolics in wines, spirits, and vinegar requires the use of sample preparation and purification stages. A suitable method for the determination of over 50 phenolic compounds (belonging to groups hydroxybenzoic acids, hydroxycinnamic acids, hydroxyphenylacetic acids, hydroxybenzaldehydes, hydroxycinnamaldehydes, simple phenols, alkylphenols, methoxy and alkylmethoxyphenols, dimethoxyphenol and alkyldimethoxyphenols, alkylphenyl and alkylphenylmethoxy alcohols, hydroxyphenylpropenes, hydroxybenzoketones and derivatives, flavanols, and hydroxycoumarins) is the SPE technique, which can be used in the online mode. The use of online SPE with a polystyrene DVB porous sorbent with urea functional groups enables convenient analysis and elimination of matrix interferences during chromatographic determinations. Only cresols, ellagic acid, phenol, and tryptophol were challenging in analysis and characterized by a high LOQ [84].

### 3.7. Limitations of SPE for Food Analysis

There are various formats and sorbents used for SPE; however, this method also did not avoid certain limitations. Commercially available SPE cartridges, discs, and multi-well plates are relatively expensive and generate a substantial amount of plastic. Moreover, they have a limited capacity and plugging can occur [85]. And although there are many sorbents developed to date, these sorbents are also a limiting factor and SPE cannot be used for the analysis of every compound of interest.

## 4. SPME in Food Analysis—Main Fields of Applications

### 4.1. Volatile Compounds

Food matrices contain a great number of VOCs, derived from many metabolic pathways. Such chemical diversity creates difficulties in their analysis. SPME is a commonly used method for the qualitative, but also quantitative analysis of VOCs in food products. A properly optimized SPME method provides a sensitive, robust and solvent-free solution for the analysis of VOCs. SPME can give a linear response over a wide range of analyte concentrations; however, it needs to be optimized for each type of matrix separately [86]. The majority of studies used SPME to extract VOCs from the headspace; however, there is also the possibility of using SPME for direct extraction, which can be more suitable for less volatile compounds [17]. Numerous studies published in the literature showed a possibility of using SPME, mostly coupled with GC–MS for analysis of volatile markers classification of samples according to different criteria, such as geographical or botanical origin, technological processes, quality state and detection of adulteration [87,88,89,90].

A great number of articles concerning SPME as a method of choice in food analysis proved the great potential of this technique. Available articles pertain to diversified foodstuffs including plant products, meat products, alcohols and various processed foods [91,92,93,94]. Many applications of SPME in plants and plant-derived product analysis have been noted for the last 20 years. Volatiles and semi-volatiles play important roles in plant physiological processes, but they can also be different plant protection products, organic pollutants and other toxic substances which can enter the plants from sprays or the atmosphere. Due to growing awareness of health risks, monitoring changes in the VOCs profile is very important. Traditional pre-treatment methods are not always convenient for the analysis of unstable aroma substances, which can be easily decomposed thermally. Therefore, SPME in connection with GC meets all the criteria of a suitable technique for plants VOCs analysis [95]. The great potential of the technique lies in its ability to monitor VOCs in vivo in plants; however, it is another subject related more to the area of plant physiology than food analysis.

Analysis of VOCs in the sample can be targeted or non-targeted. Targeted methods are focused on the detection of one or a few defined compounds/groups of compounds, while non-targeted analysis aims to see the maximum number of compounds in the analyzed sample. To date, most of the methods applied for the investigation of VOCs in food were targeted; however, recent advances in HRMS together with user-friendly software enabled the development of non-targeted analysis. Non-targeted analysis of food with subsequent multivariate analysis produces a food fingerprint, which can be described as molecular markers or markers representing characteristic food states or conditions. A complementary approach to metabolic fingerprints is metabolic profiling, which is focused on the analysis of specific groups of compounds, related to certain metabolic pathways [96]. Metabolic fingerprints and metabolic profiling are used in metabolomics. This term describes a study of metabolites/small molecules occurring in a biological system with the aim of analyzing as many components as possible [97]. Recently, the specific branch of metabolomics focused on food has been called foodomics [98].

Food fingerprints help to answer many questions about the authenticity or quality of food [99]. Therefore, it is important to know the VOC pattern of a given food product and the divergences occurring as a result of storage or processing. Minimal sample preparation is especially important in untargeted approaches without a predetermined hypothesis; therefore, SPME is frequently used for this purpose. However, it has to be remembered that the concentration of compounds present in the headspace does not cover their concentration in the matrix. The partition coefficient of compounds presented in a sample depends on the physiological properties of the substances, type of matrix and isolation conditions.

Based on SPME–GC data, many plants or generally food products have been successfully discriminated. SPME coupled directly with MS and multivariate analysis was applied to predict the shelf life of pasteurized milk. SPME–MS was shown to be a more accurate technique than microbiological plating methods (a technique previously reported in this kind of research) [100]. CAR/PDMS fiber was used in those studies and it proved to have advantages over static headspace or dynamic headspace techniques, such as no need for specialized equipment. It was also more efficient at extracting volatile fatty acids from milk. The described system shows a strong potential application for routine work in the dairy industry [100]. Peres et al. [101] characterized aroma compounds in Camembert cheese by SPME–GC–MS. The obtained results were used in a subsequent experiment, which consisted of the direct introduction of extracted compounds into the ionization chamber of MS without chromatographic separation. The results were processed and proved to be a valuable tool in qualitative classification [102]. The SPME–MS approach, followed by statistical analysis—principal component analysis and linear discriminant analysis—was successfully applied as a rapid technique for the biological origin determination of raw spirits [103]. The presented analytical tool enabled determining the origin of samples, depending on the raw material they were produced from. Lack of chromatographic separation and fast 2 min extraction provided results after an extremely short time, which is especially important in laboratory routines [103]. The technique was also used for profiling VOCs in blackcurrant varieties depending on growth latitudes and environmental factors. The study showed the changes in the volatile fraction depending on the growth environment on the composition and quality of analyzed fruits [104].

Another field of interest regarding the implementation of SPME is research on food authenticity. The adulteration of juices has become a significant issue in the beverage industry. SPME and GC with a flame ionization detector (FID) were used in the study by Ruiz del Castillo et al. [105], who assessed the adulteration of juices as a tool for enantiomeric purity evaluation. The enantiomeric composition of certain chiral terpenes was investigated to determine the origin of aromas as well as their addition to fruit beverages. The authors concluded that the enantiomeric distribution of chiral terpenes can be a valuable tool in the detection of aromas in addition to fruit beverages. Alteration in enantiomeric distribution may also be an indicator of falsification [105]. The next food product, the authenticity of which is extensively investigated due to many sophisticated adulteration techniques is honey. Two principal techniques are involved in honey falsification: addition to water and extension with sugar/syrups and feeding bees with sugar or syrup labelled as those kinds of honey with the fake floral or geographical origin [106]. VOCs in honey samples contribute to its flavor, and also to the differentiation of honey with floral origin and method of handling [107]. Evaluation of meat and dairy VOCs is of increasing concern, mostly due to the development of sensors informing consumers if meat is spoiled or not. To create this kind of sensor, numerous studies have to be conducted to decide which compounds are indicators of meat spoilage. SPME meets expectations essential for the technique used for this purpose. So far, it has been widely applied in research focused on VOCs occurring in spoilage of food. Recent studies reported the production of VOCs by bacteria isolated from different foods.

### 4.2. Flavor Compounds

The term flavor is assumed to be the interaction of the taste, odor and textural feeling of a food product. It is perceived mostly by the sense of smell and taste. Therefore, flavor compounds can be divided into two classes: compounds responsible for taste and aroma; however, there are substances providing both sensations. Taste compounds are generally non-volatile, whereas aroma compounds are always volatile. Odors are characterized by relatively high vapor pressures and their molecular weight does not usually exceed 300 Da to be detectable by the odor receptor sites in the nasal cavity. SPME is a technique most often used for the analysis of VOCs, as well as many studies on flavor for aroma compound analysis.

The number of VOCs detected in foods exceeds 10,000—among them, only a few hundred have aroma activity [108]. They usually occur at extremely low amounts in food matrices and consist of many various organic compounds possessing different polarities and reactivities. Moreover, food flavor compounds are challenging to analyze due to their very low concentration and bounding into the food matrix. Commonly used exhaustive extraction methods such as solvent extraction, steam distillation extraction (SDE) or SAFE require a long preparation process before chromatographic analysis. The aroma compounds are also analyzed by direct headspace sampling or purge and trap methods. However, those techniques are expensive and often suffer from low sensitivity.

Certain applications of SPME in flavor research can be distinguished: comparison of different plant cultivars, profiling of particular food products, analysis of compounds important for food quality, monitoring of technological processes on aroma, monitoring of chemical/biochemical processes on flavor substance degradation and use of SPME for GC-olfactometry purposes [109]. Different matrixes were analyzed using SPME, including plant [110], meat products [111], dairy products [112] and beverages [113].

The first attempt at SPME application in flavor analysis was made by Yang and Peppard in 1994 [114]. They tested SPME for 25 common flavor compounds in water solutions as well as for authentic samples like juice or coffee. It was proved that any changes in extraction conditions strongly affected the absorption distribution. SPME was found to be a useful technique in flavor analysis, mostly as a complementary tool to traditional extraction techniques. According to the authors, the main disadvantage of this technique was problematic quantitative determination [114]. Nowadays, the development and availability of isotope-labelled standards enables overcoming those limitations. However, to quantify VOCs, the appropriately optimized method and appropriate internal standards have to be selected [86,115].

In a study conducted by Roberts et al. [116], different SPME fibers were compared for their utility for aroma compound analysis. PDMS/DVB fiber was the most sensitive for heavier polar odorants like vanillin. Carboxen/PDMS was the most suitable for small molecules such as 2-methylpropanal and acetaldehyde. Carbowax/DVB was found to be adequate for organic acids. Non-polar substances were detected at the ppb level, while polar substances were more problematic in analysis. Polar compounds of low volatility such as sotolon and vanillin had a meaningful impact on the overall aroma. It was observed that SPME has difficulties detecting those aromas below the ppm level.

Off-flavor analysis is a field of high interest to scientists and food producers. Compounds that caused off-flavor or food taints mostly belong to the following groups: sulfur compounds, alcohols, fatty acids, haloanisoles, chlorophenols, phenolic compounds, esters and amines. A few basic steps are involved in their identification—first, gathering information about the analyzed product origin, subsequently extraction and concentration of VOCs, GC-O and GC–MS to, respectively, select and identify compounds responsible for unpleasant odors and finally spiking the analyzed product with the identified compound to confirm the obtained results [117]. Sulfides are widely distributed in both animal and plant products and they are quite problematic for instrumental analysis [118]. Apart from low concentrations that occur in foodstuff, the main issue is their reactivity taking place during isolation as well as during injection on a chromatograph. It was observed that thiol oxidation was minimized during sampling. CAR/PDMS was found to be the most adequate fiber for their analysis; due to their high volatility, the best results were observed after extraction in low temperatures [119].

Many studies about key aroma compounds in selected food products involve SPME, but mostly as an addition to exhaustive extraction techniques like SAFE and SDE [120]. SPME usually is less efficient in the extraction of aroma compounds than exhaustive techniques. In contrast, SPME was found to be more sensitive to esters and highly volatile compounds than SAFE isolated from apple cider [121]. The authors claimed that both techniques should be used to provide a more complete aroma profile [121]. Majcher and Jeleń [122] compared the suitability of the SAFE, SPME and SDE methods for the isolation of flavor compounds from extruded potato snacks. According to the authors, SPME itself is not enough for full characterization of potato snack aroma, mostly due to the absence of VOCs of higher weight. An exhaustive technique like SAFE was necessary for a full characterization of aroma-active compounds in the matrix. Sniffing the extract from SPME did not reveal more than a few important compounds; however, this technique was very precise, sensitive and suitable for the analysis of low-boiling compounds [122]. Moreover, SPME is also important for identifying compounds in the first few minutes, hidden under the solvent peak on the GC–MS chromatogram. The extraction of VOCs from watermelon juice allowed for the identification of 40 aroma-active compounds—among them, 30 were identified by SPME and 28 by SAFE. SPME was better for the extraction of aldehydes and alcohols, while SAFE showed better effects in sulfur extraction [123]. Nevertheless, in studies dealing with the determination of key aroma compounds in certain food products, SAFE remains the preferred technique. The application of a low temperature in combination with a high vacuum during the procedure led to obtaining a high-quality extract. The extract is enriched with VOCs of different polarities and a broad range of molecular masses. Moreover, a sample can be frozen and reused, which is especially important during working with fresh material, the chemical composition of which can change significantly during storage.

In flavor research, aroma–food matrix interactions have aroused special attention. VOCs presented in the headspace are particularly interesting because they can travel to the nose receptors during chewing [124]. An exhaustive isolation technique would not enable the inclusion of the effect of food components (like protein, lipids or carbohydrates) on the partition coefficient of aroma substances, while headspace techniques (including SPME) are able to show how matrix impacts the release of aromas.

To date, most available research treated SPME as a valuable tool for qualitative analysis, and a minority used it for quantitative purposes. Its sensitivity, selectivity and reproducibility make it an adequate tool for quantitative analysis. The utility of SPME in aroma studies is especially visible in the targeted analysis of odorants, rather than research focusing on the determination of key aroma compounds. SPME extract is a relatively poor representation of the real product aroma. It is mostly caused by differences in recovery rates, depending on the chemical properties of VOCs and partition coefficients occurring during the procedure.

### 4.3. Processing Contaminants

Safety is generally one of the main goals of food analysis. Many efforts have been made to ensure the highest quality of reconstituted products. Food processing, like cooking, fermentation, packing, transport or storage, is a significant source of contamination. Thus, it is important to monitor food at each step of the production process. The amounts of hazardous substances are regulated by appropriate institutions, which describe the minimum level recognized as safe for humans.

Cooking is a well-known process that causes substantial changes in food stuff, and some common contaminants such as heterocyclic amines, acrylamide or PAHs can be formed at a higher temperature. All the mentioned compounds were determined in food products by the SPME technique. Cardenes and co-workers [125] used SPME coupled with HPLC with a DAD for the analysis of heterocyclic aromatic amines. The authors compared four different fibers for the extraction efficiency of heterocyclic aromatic amines from beef extracts. CW-TPR fiber was recommended as the most suitable, enabling the determination of heterocyclic amines at low ng/mL levels. The only difference between SPME–GC and SPME–HPLC is the desorption step. An HPLC-specific interface with an appropriate fiber-desorption chamber is required. Through the interface, the mobile phase contacts the SPME fiber, and the absorbed compounds are removed and flow with the mobile phase to the column [126]. Acrylamide is a very common processing contaminant, occurring in starchy products under the influence of high temperatures (above 120 °C). In 2015, the EFSA published a full risk assessment of this substance present in food. Experts concluded that it potentially increases the risk of cancer development. SPME coupled with GC–MS/MS was used for the direct determination of this toxic compound in aqueous matrices [127]. The obtained LOD was 0.1 µg/L. The proposed method was successfully applied for the quantification of acrylamide in products like French fries or potato chips.

Another example of the application of SPME was the determination of furan in baby food samples. This compound is regarded as a possible carcinogen (group IIB) by the International Agency for Research on Cancer. The high volatility of furan causes its determination to be challenging. SPME followed by GC–MS was developed and gave satisfactory results in different matrices. The amount of furan analyzed in baby-food samples varied between 4.7 and 90.3 µg/kg. It was proved that the technique was appropriate for reliable determination of furan. Obtained LOD and LOQ parameters were, respectively, 1.9 µg/kg and 4.0 µg/kg [128].

3-monochloropropane-1,2-diol (3-MCPD) is considered a possible human carcinogen and the European Commission Scientific Committee on food determined 2 g/kg body weight as a safe daily intake. Determination of extremely low quantities of 3-MCPD in the food matrix poses a challenge for analytical chemists despite impediments such as a high boiling point or low molecular weight negatively affecting separation by GC–MS. This technique became commonly applied in analytical laboratories; however, the substance requires derivatization before analysis to overcome the problem of low volatility and high polarity. Derivatization also solves the problem of low molecular weight, which makes it difficult to distinguish the 3-MCPD from background noise. The trend of looking for new techniques is focused on the elimination of liquid extraction and labor-intensive steps in sample preparation, ideally following green chemistry principles. Meeting these requirements, SPME–GC–MS was developed for 3-MCPD determination in liquid hydrolyzed vegetable protein and soy sauce. The LOD was 3.78 ng/g [129]. Later, Lee and colleagues [130] used the technique to determine 1,3-dichloro-2-propanol and 3-MCPD in soy sauce samples and obtained an LOD for 3-MCPD 4.62 ng/mL.

Analysis of contaminants in food is second to that of flavor compounds as the most expanding field of SPME application in the food industry; however, its utilization is mostly related to volatile or derivative VOCs. There are relatively few works describing SPME followed by LC separation. With increasing concern about food safety and possibilities created by SPME, the scale of research could be increased by real-time monitoring (both in vivo and in vitro), accumulation and metabolism of contaminants.

### 4.4. Limitations of SPME in Food Analysis

Similarly to SPE, SPME has some limitations. The SPME fibers are not uniformly sensitive to all compounds, thus peak areas for an SPME sample do not properly reflect the true proportion of VOCs in the headspace. Moreover, the adsorption selectivity of the fiber and competition between VOCs in their adsorption of the SPME fiber may affect quantification [131]. A recent commentary underlines the problem of competition on the fiber, including internal standards, which can lead to inappropriate “semi-quantification” [115]. The food matrix is in general rich in polar compounds, which have lower affinity to the coatings used for SPME fibers. This can lead to saturation, swelling and displacement effects during the analysis of VOCs in food products [2]. Another limitation of SPME is the high cost of fibers, which additionally are very fragile and easy to break. Some attempts have been made to diminish this limitation by the production of more resistant stable-flex fibers and SPME fibers on metal alloys instead of silica, which improves the mechanical stability of the device [132].

### 4.5. Advances in Geometries of SPME

**Figure 2:**
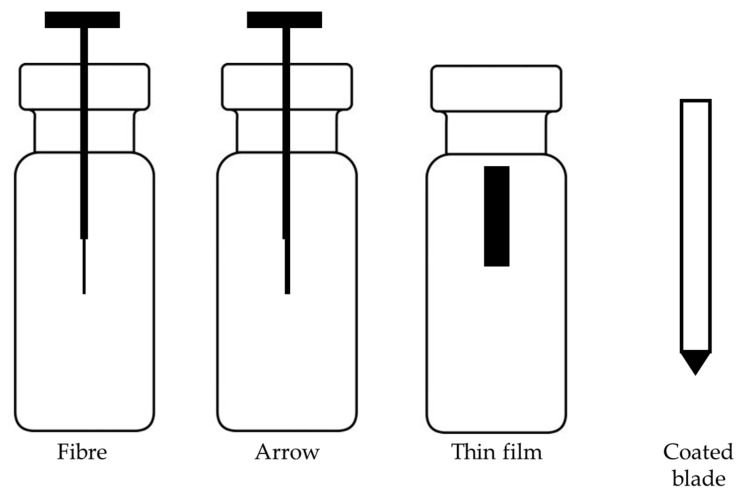
Different geometries of SPME.

The traditional SPME consists of coated fused silica fiber. This device, developed in the early 1990s, has certain limitations described in the previous section. To overcome these limitations, improvements and new geometries of SPME were proposed in recent years, as presented in Figure 2. The main direction of new developments was increasing the surface area of the coating to increase the extraction rate. An example of a device with an increased surface area is SPME arrows, which are based on a stainless-steel rod fiber with an arrow tip [133]. Compared to fibers, arrows improve the robustness, help to avoid background contamination and have higher sensitivity due to the possibility of longer and thicker sorbent phases [134]. This leads to the limitation of this device, which is the longer extraction time needed to reach equilibrium. Another drawback of arrows is the need to modify the GC inlet with specially designed liners with higher diameters compared to SPME fibers. SPME arrows have been successfully applied for the analysis of compounds in food matrices, including fermented fish sauce [135], Korean distilled spirit [136] and Korean edible plants [137]. Thin-film solid-phase microextraction (TF-SPME) has been validated as a novel sampling device and it demonstrated potential as an extraction method used in food analysis. TF-SPME is characterized by a larger extraction phase volume and surface area-to-volume ratio compared to traditional SPME fibers [138]. Therefore, TF-SPME enables enhanced capacity and a higher extraction rate than the traditional form. This format of SPME was applied in combination with LC, GC, and direct MS. Coated blade spray (CBS), which is a sorbent spread on the metal plates coupled directly to the MS device, was successfully used for the analysis of 1–5 veterinary drugs in meat [139]. A sorbent spread on aluminum foil was used for the determination of five individual polychlorinated n-alkanes from cod liver oil samples [140]. Recently, sorbent spread on carbon mesh was also used as an extraction tool for odorants from beer [2]. TF-SPME demonstrated a high potential for food analysis since it enables analysis of a wide range of compounds, and more varieties of sorbent phases have been utilized in this form of SPME [2]. The only drawback of this device compared with SPME fibers is the lack of full automation. Although the desorption of TF-SPME might be fully automated, for both LC and GC, their geometry still poses a barrier for online extraction.

## 5. Other SBE Approaches in Food Analysis

Although SPE and SPME prevail as the sample preparation steps among SBE methods in food analysis, they are not the only ones that are used. SBSE is probably the most often used apart from the aforementioned methods.

### 5.1. Stir Bar Sorptive Extraction

**Figure 3:**
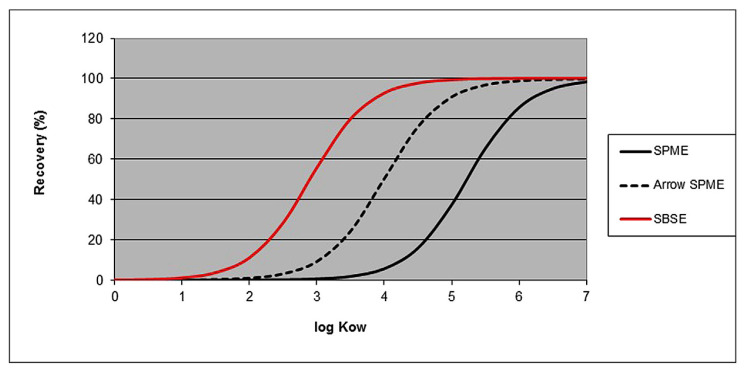
Theoretical recovery in the function of log K_ow_ for three sorptive extraction methods (V_sample_ = 5 mL; V_PDMS_ = 0.6 µL, 10.2 µL and 126 µL for SPME, Arrow-SPME and SBSE, respectively. Based on [143].

SBSE was developed in 1999 based on the observation of absorption/adsorption phenomena on PDMS, introduced as magnetic stir bars coated with PDMS and almost instantly commercialized as Twister by Gerstel [141]. Since the development of the technique, it has gained high popularity in various aspects of environmental, pharmaceutical, biomedical and food and flavor extractions. Many reviews summarized the developments in SBSE, with the first 10-year period thoroughly described by Prieto et al. [142] and the developments of recent decades described by David et al. [143]. The sensitivity obtained by SBSE, which made it the preferred technique for many trace analyses, is related to the volume of PDMS compared to other techniques in which PDMS is used as an absorbent (i.e., SPME), though the desorption of analyte from such a high-volume (and thick layer) sorbent also poses problems for carry over and incomplete desorption of compounds absorbed into PDMS (memory effect) [144]. The benefits of SBSE compared to other techniques are presented in Figure 3, which shows the comparison of theoretical recoveries of analytes as a function of the octanol/water partition coefficient (log K_ow_). It is evident that, especially for compounds with lower log K_ow_, SBSE is superior to SPME or Arrow-SPME (which operates similarly to SPME but has more sorbent) in terms of extraction yield.

The idea behind SBSE makes it ideal for trace analysis; therefore, it found numerous applications in food analysis [145]. SBSE in the direct extraction mode is used for the extraction of analytes from liquid foods; for solid foods, ultrasonic extraction is sometimes used. In food analysis, SBSE is used in fields where high sensitivity is required, i.e., for trace analysis of contaminants such as pesticides, PAHs, phenols furans, volatiles and compounds responsible for off-odors. PDMS-coated stir bar extraction is similar to LLE using a polar solvent; therefore, this approach is used for the extraction of contaminants from non-fatty matrices, such as juice, wine, fruit and vegetables [146]. The main disadvantage of SBSE in food analysis was the lack of various extraction phases, with a polar PDMS used in the majority of cases. In food analysis, the extraction of polar compounds at trace levels is a challenging task. The difficulties in the extraction of polar compounds from food matrix often result in their under-representation when, for example, aroma compounds are profiled in foods [2]. The situation in SPME is better due to the higher number of fiber coatings of different polarity available; however, SBSE offers higher compound capacity due to its much higher phase volume than SPME. SBSE phases for polar compounds have been developed; however, their robustness and stability are low and they were used mostly with liquid, not with thermal desorption. The most popular alternative coating is polyethylene glycol-modified silicone (EG). This can be used with thermal desorption, though its durability is still lower compared to PDMS. SBSE with EG was used for the extraction of volatile phenols from alcoholic beverages as the limited affinity of PDMS to polar compounds makes their extraction (especially for 4-vinylphenol) unsatisfactory. Low LOQs (<3 µg/L) make it an attractive method considering the influence of these compounds on the aroma of alcoholic beverages [147]. An alternative approach for the analysis of polar compounds (K < 3.0) in aqueous samples is their derivatization, which aims at lowering the polarity of analyzed compounds and increasing the recoveries of the derivatives. Such an approach is usually performed for target analysis of thiols or carbonyl compounds in alcoholic beverages [148]. Similarly to SPME strategies, for SBSE, the derivatization process can also be performed in situ on a stir bar and post-extraction with specific derivatization reactions employed: alkylation, acylation or silylation (in this case, usually post-extraction due to the sensitivity of silylation agents to water). Ochiai et al. [149] summarized several new approaches in the analysis of polar compounds by SBSE in aqueous solutions. They involve SBSE with freeze concentration (called ICECLES), which is performed in a dedicated device which freezes the solution during extraction, enabling a gradual concentration of solutes in the remaining liquid part, whereas pure water freezes during the extraction process. Another proposed approach is Solvent-Swollen PDMS (also called solvent-assisted SMSE, SA-SBSE), in which the solvent (such as dichloromethane, diethyl ether, and diisopropyl ether) acts as a modifier to PDMS, but also as an additional extraction medium. It was found to be suitable for the extraction of compounds with low K_ow_ values, such as 2-acetyl pyrrole or indole from water, and was used for aroma compounds in wine [149]. Nowadays, the developments in the SBSE technique for food applications aim to widen the extraction efficiency for compounds of various polarities. Such an approach is reflected in the use of dual SBSE. Due to the different polarity of extracted pesticides, a dual SBSE method was proposed with simultaneous desorption of two stir bars used for extraction from different solvent systems (one of them modified with the addition of NaCl). A comprehensive review of novel approaches and sorbent technologies for SBSE was written by He et al. [146], where examples of extraction modes for the simultaneous extraction of multiple compounds with different polarities are summarized. For such cases, apart from dual SBSE, multi-shot or sequential modes of SBSE can be used to avoid the decreased sensitivity resulting from the extraction of analytes with different polarity.

Although compared to SPME, SBSE suffers from a lack of a broad range of extraction phases, developments in new phases for SBSE are also reflected in food analysis: experiments were performed with C18 coatings for sulfonamides in milk, cation exchange monoliths for cations in milk, PDMS/activated carbon for pesticides in sugarcane juice, MIPs in triazine herbicides in rice, apple, and lettuce, and TiO_2_-PPHF for arsenic species in chicken tissue, to name the most interesting [145]. Experiments were also performed for novel phases for SBSE such as polypyrrole, polyacrylate, or polyurethane (PU) foams. The latter were used for the analysis of pesticides in water and coffee, but also hormones and acidic pharmaceuticals [150]. Many techniques used for sorbent production are applied to SBSE coatings manufacturing at a lab scale including sol–gel technology, monolithic technology, molecularly imprinting technology and physical adhesion methods [146]. Dual-phase SBSE using two differently coated stir bars (OH-TSO-coated stir bar and C18—PDMS coated stir bar) was proposed for the extraction of preservatives of different polarity, and the sequential SBSE mode was used for the analysis of pesticides extracted under optimal conditions sequentially and desorbed in one GC–MS run. In the multi-shot mode, different sample aliquots are extracted under the same or different extraction conditions using one coated bar in each aliquot, and then the stir bars are simultaneously desorbed, whereas two stir bars are used in dual SBSE to extract two aliquots of a sample under different extraction conditions before both stir bars are simultaneously desorbed for the subsequent analysis. Dual-phase SBSE is performed in one aliquot of sample solution whereas two or more stir bars are used sequentially in sequential SBSE for one aliquot under two or more different extraction conditions [146]. The main mode of extraction for SBSE is a direct extraction from aqueous solutions. It was found to be suitable for the analysis of alcoholic beverages, due to the low affinity of PDMS to alcohol and very good efficiency in the extraction of various aroma compounds, and compounds responsible for off odors in alcoholic beverages. On the other hand, especially in the analysis of aroma compounds in food and plant material, extraction was proposed to be performed from headspace [151]. The extraction of aroma compounds from headspace using stir bars is called headspace sorptive extraction (HSSE) and has found numerous applications in aroma analysis, especially for analysis of aldehydes, terpenes, and alcohols esters, in various food and plant matrices such as fungi, olive oils, wines, vinegar, cheese and garlic [146]. The term high-capacity headspace sorptive extraction (HCSE) is often used synonymously with HSSE, though a stir bar is usually used in the latter approach for the extraction of VOCs from the headspace, whereas HCSE used another type of extraction device when first described. Tienpont et al. [152] proposed a glass rod covered with PDMS (50 mg PDMS was deposited on a rod of 1 mm i.d.). The rods were placed in the desorption tubes after extraction and subsequently desorbed. A high enrichment factor for highly volatile compounds was achieved, which resulted in the high sensitivity of the proposed method used for monitoring VOCs in bananas or coffee [152].

SBSE was used for the extraction of fruit VOCs in the investigation of the enantiomer composition of aroma compounds. Kreck et al. [153], who used SBSE for the extraction of VOCs from the strawberry pulp after homogenization with 20% NaCl, used it in the HSSE mode for the extraction of aroma compounds from intact fruit, but also performed an “in fruit” sampling procedure, squeezing a stir bar into an intact fruit for a 3 h extraction. The enantiomeric composition of crucial odorants was performed after thermal desorption into a multidimensional GC–MS system [153]. Another interesting application is the use of SBSE for the in vivo extraction of VOCs released in the mouth during food consumption [154].

### 5.2. Molecularly Imprinted Polymers (MIPs)

**Figure 4:**
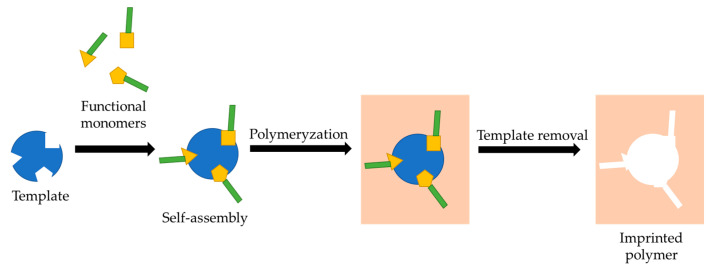
Manufacturing of MIPs.

For sorbent-based methods, the lack of selectivity of sorbents is the main drawback. In research on selective sorbents, different materials have been tested, such as restricted-access materials (RAMs), immunosorbents or oligosorbents. RAMs, which interact via hydrophobic interactions and a size exclusion effect, found the majority of applications in toxicological and clinical analyses, immunosorbents and oligosorbents synthesized using antibodies and aptamers, respectively, bound to solid support also found numerous applications in food analysis (i.e., immunoaffinity columns for mycotoxins analysis) [21]. The main area in selective sorbent phases, developed since the 1990s, is the construction of MIPs—synthetic materials able to bind target molecules mimicking natural receptors. Manufacturing involves three steps: (i) formation of the pre-polymerization complex between the template and the monomer; (ii) polymerization in the presence of a cross-linker; (iii) removal of the template to give the imprinted polymer (Figure 4) [21]. The analytical potential of MIPs is also related to their commercial availability, where MIPs tailored for analysis of, i.e., specific mycotoxins or bisphenols, are available. In the majority of applications, MIPs are designed for SPE; consequently, molecularly imprinted solid-phase extraction (MISPE) can be realized in two modes: offline and online. The offline process requires the same approach to SPE and the eluate can be analyzed by GC or HPLC, which is automated in the direct coupling of MIPs in the analytical system [155]. MIPs found applications mostly in the analysis of harmful substances in food, such as pesticide residues, veterinary drugs, dyes or toxins. The detailed discussion of the application of MIPs in the detection of the main regulated groups of contaminants has been discussed by Regal et al. [155]. MIPs are used for the analysis of residues in food which are classified by EU Council Directive 96/23/EC: drugs as growth promoters (anabolics, antithyroid agents, beta-agonists), authorized veterinary drugs and contaminants. MIPs have been used for the analysis of stilbenes in fish and milk, β-agonists in pork chloramphenicol in milk and honey [156,157]. The majority of them were analyzed in MISPE with HPLC to separate the compounds. Similarly, tetracyclines were analyzed in fish, eggs, and chicken muscle using MISPE, but also MIPs on SPME fiber [158]. MIP-SPME mentioned here used for the analysis of tetracycline and related compounds oxytetracycline, doxycycline and chlortetracycline offered LODs in the range of 1–2.3 µg/L when analyzed by HPLC with a fluorometric detector. Quinolones and fluoroquilnolones were determined by MISPE and HPLC in fish, chicken muscle and baby foods. The use of MIPs for d-SPE was also explored for applications in food analysis and used for the detection of dioctyl phthalate in fish [159] and acrylamide in bread [160].

A summary of MISPE and MISPME was presented by Song et al. [161], with information on herbicides, contaminants, and pharmaceuticals in food samples but also trace metals such as copper ions, selenium and arsenic. Among the 60 papers shown, 4 utilized SPME for MIPS, and the remaining papers mostly used MISPE. Magnetic solid-phase extraction (MSPE) can also benefit from MIPs and procedures were developed for preparing MIPs for the analysis of 17β-estradiol in milk, FQs, sulfonamides in chicken breast, curcumin in various foods, malachite green in fish, PCBs, phthalates or mycotoxins [21]. Although MIPs are usually used with solid-phase material and HPLC is usually utilized for compound detection, it can be also used in other approaches as described by BelBruno et al. [162]. Quartz crystal microbalances (QCMs) were used for the determination of histamine in foods, with a histamine-targeted MIP coated onto the QCM with an LOD of 10^−4^ mg/kg achieved. A MIP sensor for melamine detection in water and milk was also constructed with surface-enhanced Raman spectroscopy used for detection. Other uses of MIPs incorporated into ZnS quantum dots, and conjunctions with differential pulse voltammetry as a detection method have also been described [162].

### 5.3. Needle Traps

Needle-trap devices comprise a group of techniques in which the sorbent is placed on a needle wall, or fills the needle. When mounted to a syringe, a repetitive plunger movement will induce airflow (headspace flow) along/through the sorbent layer [163].

One of the most established techniques for analysis of VOCs is static headspace developed at the beginning of the 1960s; though in an automated form, very convenient, robust and easy to perform, it suffers a main disadvantage—relatively low sensitivity due to a lack of preconcentration. A certain volume from headspace over the sample (i.e., 1 mL) is transferred by a gas-tight syringe or loop system into the GC. To enhance the sensitivity of static headspace, multiple headspaces with trapping analytes in a special Tenax trap can be performed in special types of headspace samplers in which, after the several extractions after which analytes are adsorbed, they are desorbed into the GC [164]. A trap is located in a separate part of the autosampler for static headspace, so it cannot be regarded as a needle trap. On the other hand, needles with PDMS coating inside have been proposed for the extraction of analytes from a headspace vial (HS-SPDE, headspace solid-phase dynamic extraction). The principle of the method is a non-equilibrium extraction similar to dynamic headspace. Therefore, for optimization of extraction conditions, several parameters need to be optimized to maximize recoveries, including sample temperature, the number of aspiration cycles, plunger speed, volume aspired, and total volume of sampled headspace. The possibility to automate and provide high-concentration factors is a bridge between static and dynamic headspace techniques [165]. A similar approach commercialized in 2006 under the name ITEX (In-Tube Extraction) differs from the aforementioned approach in the type and construction of the needle: a Tenax trap offers a 160 uL of sorbent volume and is usually packed with Tenax. The long needle with a trap has a heating coil, which enables the immediate desorption of adsorbed analytes [166]. The optimization procedure is similar to the SPDE discussed before [167].

### 5.4. Other SBE Solutions

**Figure 5:**
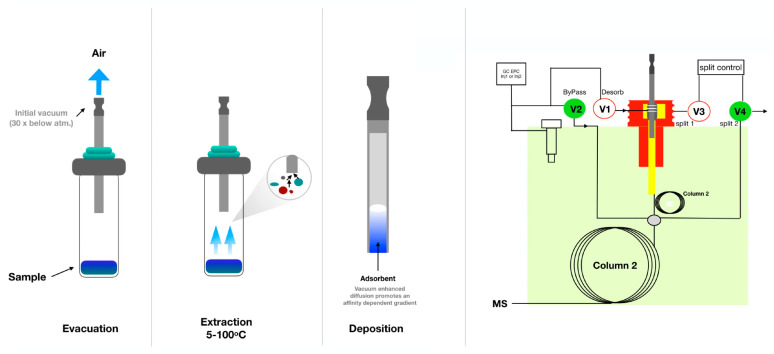
Scheme of analysis performed using vacuum-assisted flavor evaporation (VASE). V1, V2, V3, V4—valves for diverting flows during desorption process from sorbent pens.

Apart from developments in sorbents, coatings and devices for extraction, there are also developments in extraction conditions. Until recently, little attention was paid to the potential benefits of a vacuum used in sorbent extraction. Although such possibilities were indicated by Brunton et al. [168] in 2001, who used a vacuum to achieve a 4–70-fold increase in the recovery of aldehydes and alcohols in turkey breast using SPME, this fact went unnoticed for 10 years when work on the use of a vacuum in SPME was reinitiated by Psillakis et al. [169] with a theoretical model explanation and design of special vials and vessels to sample both liquid and solid matrices. A vacuum can be especially useful for compounds with a low Henry’s constant and low volatile compounds that are difficult to analyze by conventional SPME, such as organic acids and phenols [170]. Despite the majority of developments in the use of a vacuum related to SPME, devices which use a vacuum for extraction on Tenax traps (sorbent pens, commercialized by ENTECH) have recently appeared. The idea behind vacuum-assisted sorbent extraction (VASE) is to use special caps with traps with sorbents that enable air evacuation from the vial; and after extraction, the trap (resembling in size and shape GC liner, Figure 5) is inserted into a special injection port of GC, which enables rapid desorption (the same way as for sorbent tubes). Similarly to vacuum SPME, this approach improves the extraction of semi-volatile compounds. VASE was successfully applied for the analysis of terpenes in coriander oil [171] and phenols in beer [172].

## 6. Conclusions

Sample preparation methods for food analysis are rapidly evolving. Fundamental developments in extraction techniques rapidly find applications in the field of food analysis. The reason for that is the importance of food control in international trade, manufacturing and our society. Due to intensive agriculture and food production, pesticide residues, veterinary drugs, and environmental contaminants, mycotoxins need to be carefully controlled. SBE gives great analytical potential for the analysis of various compounds in food products, due to various techniques, geometries and sorbent types, which can be tailored for a specific purpose. In this review, SBE techniques, including the most commonly used, like SPE and SPME, as well as SBSE, MIPs, HCSE and NTD have been introduced and their potential and limitations were presented. The limitations of each method have to be kept in mind and the appropriate optimization of the extraction needs to be applied before the analysis to limit the risk of overestimation. The directions of future studies will most probably be focused on the development of new sorbents, simplification and automation of the process and shortening of the analysis time, i.e., by omitting the chromatographic separation step. The green character of each method has to be kept in mind; therefore, future studies will probably focus mostly on microextraction techniques, which do not consume large volumes of solvents.
